# Effect of Passive Pile on 3D Ground Deformation and on Active Pile Response

**DOI:** 10.1155/2014/904186

**Published:** 2014-08-27

**Authors:** Bingxiang Yuan, Rui Chen, Jun Teng, Tao Peng, Zhongwen Feng

**Affiliations:** Shenzhen Graduate School, Harbin Institute of Technology, Shenzhen 518055, China

## Abstract

Using a series of model tests, this study investigated the effect of a passive pile on 3D ground deformation around a laterally loaded pile and on that laterally loaded pile's response in sand. The active pile head was subjected to lateral loads, and the passive pile was arranged in front of the active pile. In the model tests, the distance between the two pile centers was set to zero (i.e., a single pile test), 2.5, 4, and 6 times the pile width (*B*). The 3D ground surface deformations around the active and passive piles were obtained using a newly developed Stereo-PIV technique. The experimental results showed that the ground surface movements were restrained by the passive pile when the pile spacing was less than 6*B*. The response of the active pile was affected by the passive pile when the pile spacing was less than 4*B*. This study combined the response of the active pile and surrounding 3D ground deformation to investigate the effect of the passive pile, which is useful to further understand the pile-soil-pile interactions and to enhance pile foundation design in engineering practice.

## 1. Introduction

Pile foundations not only transmit axial loads to the soil but also resist horizontal loads from earth pressures, wind, waves, earthquakes, and so forth. The capacity of a laterally loaded pile has been studied for more than a half century. Many methods have been proposed to analyze laterally loaded piles, such as Broms' method [1], the* p-y* curve approach [2, 3], and the strain wedge method [4]. In engineering practice, piles are most frequently used in groups. Therefore, Reese and Impe [5] and Poulos [6] conducted laterally loaded tests to study the effects of pile group and interaction among piles. Rollins et al. [7] performed laterally loaded tests and concluded that there is no group effect for spacing more than 5.65 pile diameters. Mostly, pile-soil-pile interaction was investigated for only one engineering structure by applying lateral loads at the pile cap. However, it is worth noting that the response of a laterally loaded pile is also influenced by neighbouring structures. Specifically, as the pile foundations of neighbouring structures get closer and closer due to limited urban land use, it becomes increasingly necessary to study the effect of an adjacent pile foundation on a laterally loaded pile. In addition to the response of a laterally loaded pile, surrounding ground surface deformations are generally studied. Recently, particle image velocimetry (PIV) has been used to obtain two-dimensional (2D) ground surface deformations surrounding a laterally loaded pile [8]. Using the PIV technique a 2D displacement field is obtained by correlating two consecutive images. Because of its simple implementation and accurate measurement, PIV has been utilized in various fields after its first reported application in the 1980s [9]. At present, PIV is used in many fields such as biology [10], aerospace engineering [11], and geotechnical engineering [12].

Stereoparticle image velocimetry (Stereo-PIV) is a more advanced optical measurement method. This technique is developed to measure three-dimensional (3D) deformation using two cameras instead of one camera, as is common practice in a regular PIV system. Thus, so far, the applications of Stereo-PIV have broadened. Yuan et al. [13] measured 3D sand displacements around a single pile using the Stereo-PIV technique. However, limited case studies of Stereo-PIV applications have been reported in geotechnical engineering because of the high cost of a commercial system and its complicated implementation.

In this study, a series of scaled model tests was conducted to investigate the effect of a passive pile on response of a laterally loaded pile and surrounding 3D ground deformation in sand. The effect of the pile spacing between the passive pile and the laterally loaded pile was also studied. The 3D ground deformation around the passive and active piles was measured using the newly developed Stereo-PIV system. At the same time, the lateral deflection, bending moment, and soil resistance distribution along the active pile were derived from the strain measurement; thus, the response of the active pile can be evaluated quantitatively.

## 2. Experimental Setup and Test Procedure

### 2.1. A Pile Model Test System

**Figure 1:**
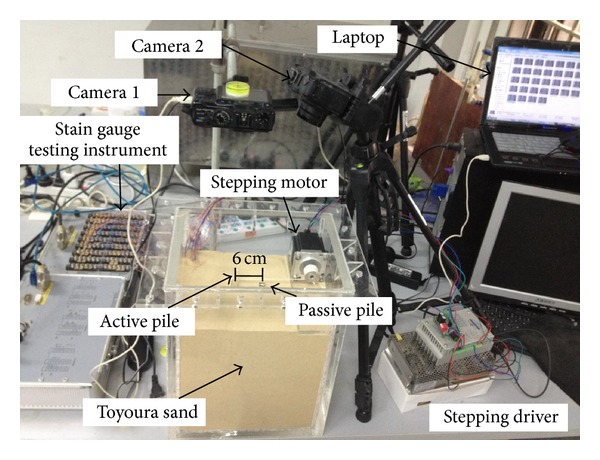
Experimental setup.

The pile model test system consisted of a model box, two model piles, two cameras, a strain gauge testing instrument, a stepping motor, a stepping driver, and a computer, as shown in Figure 1.

The model box was made of Plexiglas with dimensions of 0.20 m in width, 0.25 m in length, and 0.30 m in height. The two Plexiglas piles were square-sectioned with a width of 0.01 m, a length of 0.25 m, and Yong's modulus of 6 GPa. One pile, termed as the active pile, was subjected to lateral loads. The other, termed as the passive pile, was arranged in front of the active pile along the lateral loading direction. The lateral loading device consisted of a stepping motor with a torque capacity of 1.35 N*·*m and a stepping diver to control loading speed. To measure the bending moment, seven pairs of strain gauges were attached along the active pile. The strain gauge testing instrument was used to obtain the readings of the stain gauges.

Two cameras were used; both were Canon PowerShot G10s with 4416 × 3312 pixel resolution. They were controlled by a computer to synchronously capture images by a developed software driver using MATLAB commands [14].

### 2.2. Soil Properties

Uniform Toyoura sand was used. Its properties are summarized in Table 1. Over the past few decades, the properties and stress-strain behaviours of Toyoura sand have been extensively investigated by means of various laboratory tests, such as direct shear tests, triaxial tests, true triaxial tests, and torsional tests. The experimental data are available in the literature [15]. The relative density of the sand sample was 50%.

### 2.3. Test Procedure

Three steps were involved for one model test. The first step was to calibrate the intrinsic and extrinsic parameters of two cameras, which were set up in two optimal locations to capture images of the same region of interest. A black and white grid pattern with grid spacing of 0.01 m was targeted on the sand surface, so four pairs of pictures at different positions could be obtained to calculate the parameters of the cameras. The camera calibration toolbox for MATLAB was used to calibrate the intrinsic and extrinsic parameters of two cameras [16].

The second step was to capture a series of paired soil images and to record the data from the strain gauges. Two piles were first set in the designed position in the model box. Subsequently, the soil sample was deposited in layers by a controlled sand raining method with which sand was poured from 0.50 m height. Then, a series of paired images was consecutively captured by the two cameras when the lateral load was applied at the active pile cap. The 2D displacement vectors were calculated through two images with the demo PIVview2C analysis software [17]. At the same time, the strain gauge testing instrument was employed to obtain data from the corresponding gauges.

The last step was to compose the 3D displacement field and to derive the pile deflection and soil resistance. One sand particle movement in the global coordinate system was separately projected to two 2D movements onto the left and right camera images. On the contrary, a pair of 2D corresponding displacements can determine the 3D displacement of the sand particle. The 3D displacement vector was produced by the two camera parameters and the corresponding 2D displacement vector pairs, using the newly developed Stereo-PIV system. The bending moments were calculated from the data of strain gauges attached along the active pile. The lateral displacement and soil resistance were interpreted by double integration and double differentiation of the bending moment distribution function.

## 3. Results and Discussion

Four pile model tests were conducted with different values of pile spacing, including zero (i.e., a single pile test), 2.5, 4, and 6 times the pile width (*B*) on centre. This study combined the response of the active pile and surrounding 3D ground deformation to investigate the pile-soil-pile interaction and the effect of the pile spacing.

### 3.1. Horizontal Displacement Field

**Figure 2:**
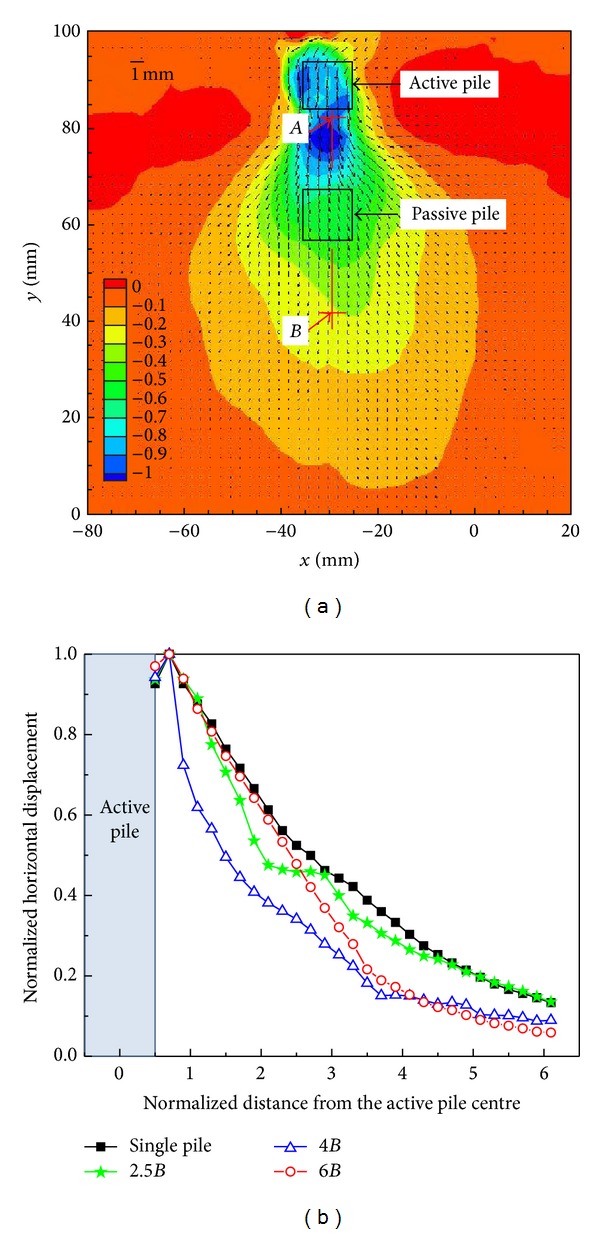
Horizontal displacement field: (a) 2D soil displacement field of 2.5*B* pile spacing; (b) normalized horizontal displacement along the section A-B in (a).

Figure 2(a) shows the planar displacement vectors and the contours of the horizontal displacements when the pile spacing was 2.5*B*. As expected, the soil moved away from the active pile during lateral loading and the soil horizontal movement was restrained by the passive pile. To explain the horizontal displacements along the loading direction at different pile spacings, the normalized horizontal displacement curves along the section A-B (depicted in Figure 2(a)) are shown in Figure 2(b), where the distance away from the active pile centre was normalized by the pile diameter and the horizontal displacement was normalized by the maximum displacement. In the curve for 2.5*B* pile spacing, the horizontal displacements were stable when the distance away from the active pile was between 2.5*B* and 3.5*B*, which was located at the passive pile. In the curve for 4*B* pile spacing, the passive pile was shown to have only a slight influence on the horizontal displacement.

### 3.2. Vertical Displacement Field

**Figure 3:**
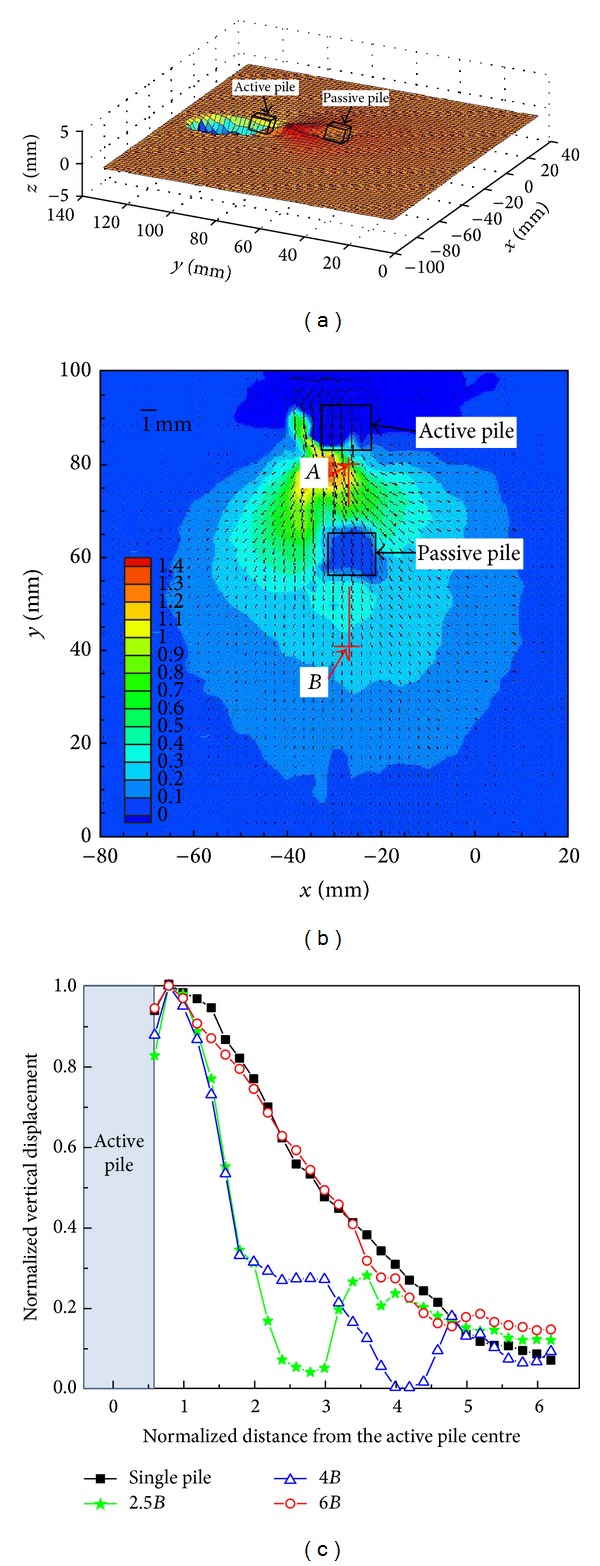
Vertical displacement field: (a) 3D view of vertical displacement field of 2.5*B* pile spacing; (b) vertical displacement field of 2.5*B* pile spacing; (c) normalized vertical displacement along the section A-B in (b).

In addition to the 2D displacement field, the vertical displacement out of planar field was also obtained by Stereo-PIV, which was easy to observe the vertical deformation around the two piles.  Figure 3(a) shows a 3D view of the vertical displacement field for 2.5*B* pile spacing.  Figure 3(b) shows the corresponding contours of the vertical displacement field. The position of the passive pile was clearly presented in Figures 3(a) and 3(b). The vertical displacements decreased with the distance away from the active pile, except at the position of the passive pile. To explain vertical displacements at different pile spacings, the normalized vertical displacement curves along the section A-B (depicted in Figure 3(b)) are shown in Figure 3(c), where the vertical displacements were normalized by the maximum vertical displacement. The vertical displacements decreased over 60% within 1.5*B* when the pile spacing was 2.5*B* and 4*B*. The curves of vertical displacement for a single pile and 6*B* pile spacing were almost overlapped, implying that the passive pile had no influence on the soil vertical displacement when the pile spacing was 6*B*. The findings showed that soil vertical movement was restrained by the passive pile when the pile spacing was less than 4*B*.

### 3.3. Lateral Load versus Displacement Curve

**Figure 4:**
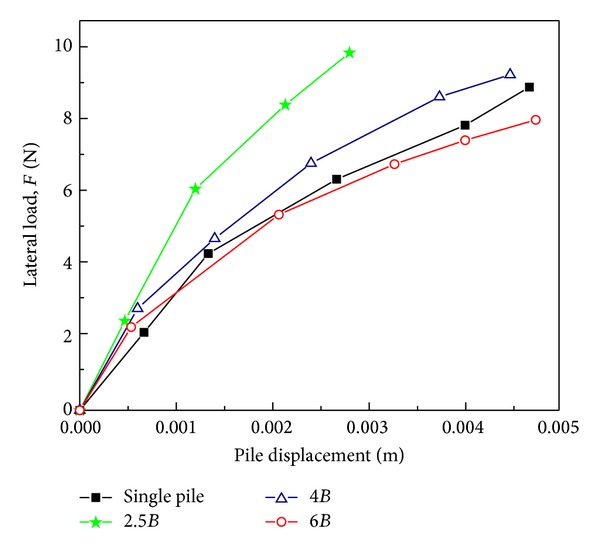
Load versus displacement curves at the active pile cap.

The curves of load versus displacement at the active pile cap for different pile spacings are shown in Figure 4. These curves were similar to the typical curve reported for a laterally loaded pile in loose sand [18]. At the initial stage, the load increased almost linearly as the pile displacement increased, which represented the elastic behaviour of soil. The rate of increasing load gradually decreased with the increasing displacement, which represented the plastic behaviour of soil. As compared with the single pile, the lateral load of the active pile at a given displacement for 2.5*B* pile spacing increased up to 40%, implying an increase in the laterally bearing capacity of the active pile due to the resistance of the passive pile. However, as the pile spacing increased, this observed effect of the passive pile gradually diminished.

### 3.4. Bending Moments along the Active Pile

**Figure 5:**
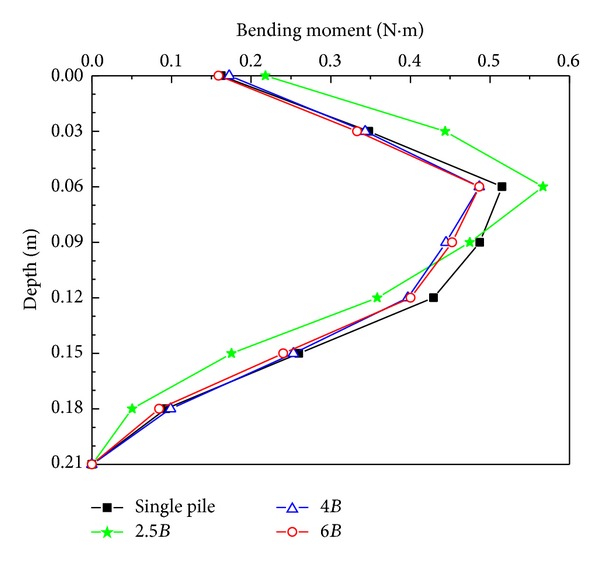
Moment versus depth curves from the active pile.

The bending moment distributions along the active pile are shown in Figure 5. Comparing the four curves, the trend of the curve for 2.5*B* pile spacing was different from the other curves. The moment data for 2.5*B* pile spacing were the largest when the depth was less than 0.06 m and were the smallest when the depth was larger than 0.12 m, indicating that the pile-soil-pile interaction for 2.5*B* pile spacing was different from the three other situations, because the passive pile resisted the intermediate soil, which in turn restrained the lateral bending of the active pile.

### 3.5. Deflection of the Active Pile

**Figure 6:**
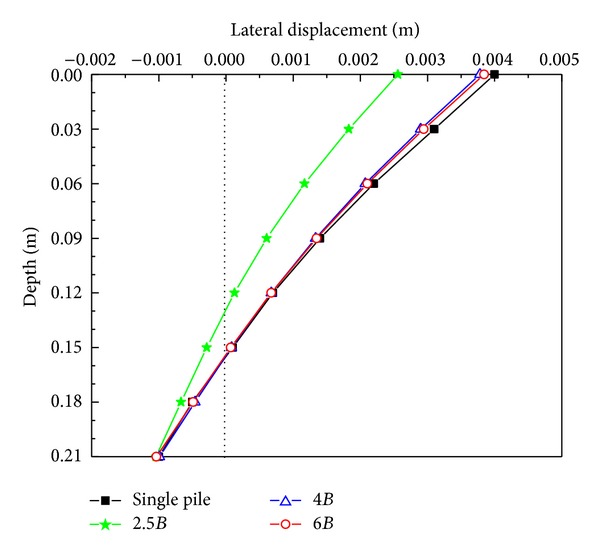
Lateral displacement versus depth curves from the active pile.

The lateral displacements along the active pile are shown in Figure 6. As mentioned earlier, the lateral load at the active pile cap for 2.5*B* pile spacing was the largest. However, the corresponding lateral displacements along the active pile were the smallest, as shown in Figure 6, because the passive pile resisted the deflection of the active pile through the intermediate soil mass when their spacing was 2.5*B*. The three other curves of deflection were almost close and this illustrated that the passive pile produced a negligible influence on the deflection of the active pile when the spacing was over 4*B*.

### 3.6. Soil Resistance

**Figure 7:**
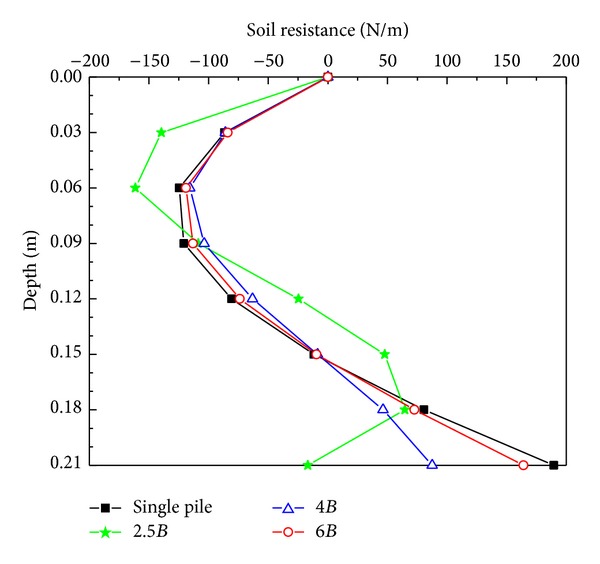
Soil resistance versus depth curves from the active pile.

Figure 7 shows the curves of soil resistance along the active pile for four pile spacings. The trend of the curve for 2.5*B* pile spacing was different from other curves possibly because of the dominant pile-soil-pile interaction. When the pile spacing was 2.5*B*, the deformation of the intermediate soil mass between the piles was restrained by the passive pile. Thus, the passive pile reacted to the intermediate soil mass and enhanced the soil resistance applied to the active pile. Therefore, the relative stiffness of the active pile decreased and it became an elastic pile rather than a rigid pile. When the pile spacing was 4*B* and 6*B*, the curves of soil resistance were close to that of a single pile, implying that the passive pile had negligible influence on the passive pile when the pile spacing was over 4*B*, which is consistent with the findings based on deflection analysis.

## 4. Conclusions

A series of pile model tests in the uniform Toyoura sand was performed to investigate the effect of a passive pile on the response of a laterally loaded pile and surrounding 3D ground deformation. Due to pile-soil-pile interaction, the induced ground movements are easily affected by the passive pile. Both horizontal and vertical displacements at the ground surface around the laterally loaded pile were restrained by the passive pile when their spacing was less than 6*B*. The deformation of the intermediate soil between the passive and active piles was resisted by the passive pile. Thus, the passive pile reacted to the intermediate soil and enhanced the soil resistance applied to the active pile. The response of the laterally loaded pile was also affected by the passive pile when their spacing was less than 4*B*.

## Figures and Tables

**Table 1:** Properties of the Toyoura sand used in this study.

Parameter	Value
Specific gravity (*G*)	2.65
Maximum void ratio (*e*_max⁡_)	0.997
Minimum void ratio (*e*_min⁡_)	0.597
Mean diameter (*D*_50_): mm	0.17
Uniformity coefficient, Cu	1.7
